# The Role of Tau Pathology in Alzheimer’s Disease and Down Syndrome

**DOI:** 10.3390/jcm13051338

**Published:** 2024-02-27

**Authors:** Ann-Charlotte Granholm, Eric D. Hamlett

**Affiliations:** 1 Department of Neurosurgery, University of Colorado Anschutz Medical Center, Aurora, CO 80045, USA; 2 Department of Pathology and Laboratory Medicine, Medical University of South Carolina, Charleston, SC 29425, USA; hamlette@musc.edu

**Keywords:** Down syndrome (DS), Alzheimer’s disease (AD), neurofibrillary tangles (NFTs), seeding competent p-Tau, neuropathology

## Abstract

**Background**: Individuals with Down syndrome (DS) exhibit an almost complete penetrance of Alzheimer’s disease (AD) pathology but are underrepresented in clinical trials for AD. The Tau protein is associated with microtubule function in the neuron and is crucial for normal axonal transport. In several different neurodegenerative disorders, Tau misfolding leads to hyper-phosphorylation of Tau (p-Tau), which may seed pathology to bystander cells and spread. This review is focused on current findings regarding p-Tau and its potential to seed pathology as a “prion-like” spreader. It also considers the consequences of p-Tau pathology leading to AD, particularly in individuals with Down syndrome. **Methods**: Scopus (SC) and PubMed (PM) were searched in English using keywords “tau AND seeding AND brain AND down syndrome”. A total of 558 SC or 529 PM potentially relevant articles were identified, of which only six SC or three PM articles mentioned Down syndrome. This review was built upon the literature and the recent findings of our group and others. **Results**: Misfolded p-Tau isoforms are seeding competent and may be responsible for spreading AD pathology. **Conclusions**: This review demonstrates recent work focused on understanding the role of neurofibrillary tangles and monomeric/oligomeric Tau in the prion-like spreading of Tau pathology in the human brain.

## 1. Introduction

Individuals with Down syndrome (DS) exhibit Alzheimer’s disease (AD)-related pathology in the brain early in life, leading to the development of dementia in their 40s or 50s, with few exceptions [1,2,3]. The AD pathology that develops in the DS brain includes widespread amyloid plaques, neuroinflammation, cell death, and neurofibrillary tangles (NFTs, [1,4,5,6]). Amyloid pathology is naturally occurring in the DS brain because the amyloid precursor protein gene (*APP*) is located on Chromosome 21 (Chr. 21), leading to increased APP and amyloid production early in life, both in humans with DS and DS mouse models [3,7,8,9,10]. Neuroinflammation is also an inherent trait of the DS brain due to several different genes encoded on Chr. 21, including four of six interferon receptors [11,12] and the superoxide dismutase 1 gene (SOD-1) [13], among other factors. Therefore, cells obtained from the DS brain exhibit early signs of inflammatory activation as well as oxidative stress [3,4,6,14,15]. 

The spreading of misfolded Tau is associated with AD and DS-AD pathology, including hyperphosphorylated Tau (p-Tau), which spreads from region to region in the brain, finally leading to a Braak stage of V-VI in the final stages of DS-AD pathology [16,17]. While AD is considered a sporadic disease of aging or as occurring earlier in life, due to the inheritance of rare genetic mutations, DS-AD presents early age-dependent kinetics of amyloidogenesis and Tau seeding, perhaps as early as the teenage years [18]. It is difficult to compare DS-AD with familial or late onset AD, since they have different biological mechanisms. This points to an absolute need for more research and clinical trials, including on those with DS, since we still do not know the mechanisms involved in the propagation of AD pathology in the Trisomy 21 brain.

We have demonstrated that neuron-derived exosomes (NDEs), isolated from the plasma of people with DS at different ages, contain unusual amounts of p-Tau; in some individuals with DS, this feature is already present in childhood [19,20]. In addition, recent work by our group has shown that NDEs from individuals with DS contain seeding-competent p-Tau that can spread to the wildtype mouse brain when NDEs are injected into the hippocampus via stereotactic injections [21]. On the basis of these findings, this review will focus on current knowledge regarding p-Tau, and more specifically its potential to seed pathology as a “prion-like” spreader leading to AD, particularly in individuals with DS.

## 2. Microtubule-Associated Protein Tau (MAPT) Structure and Function

The Tau protein is a highly conserved protein in mammals that is known to have six isoforms, which is caused by alternate splicing of exons 2, 3, and 10 of the microtubule-associated protein tau (*MAPT*) gene [22,23,24,25]. Tau is a microtubule-associated protein that contributes to the stability of microtubules in neurites and is crucial for the maintenance of normal microtubule structure and function, as well as axonal transport, in the neuron [26]. The Tau protein interacts with microtubules via either three or four microtubule binding repeats (3R or 4R, respectively), and the balance between 3R and 4R Tau proteins is altered during pathological states. When Tau undergoes modifications, this can lead to destabilization of neuronal microtubules and, consequently, to neuronal dysfunction and death [27]. Proteinaceous filaments of p-Tau are known to spread in a prion-like fashion in the human brain, leading to the detrimental formation of neurofibrillary tangles (NFTs) that contribute to AD pathology, correlate significantly with cognitive deficits in Alzheimer’s disease (AD), and contribute to distinguishing differences between AD and other tauopathies [28,29]. A distinct set of modifications occur in different neurodegenerative conditions, of which AD is the most common [26]. A precise balance between four-repeat (4R) and three-repeat (3R) isoforms of Tau are found in normal conditions, while dysregulation of the 3R:4R ratio is associated with different forms of tauopathy [23,30,31,32,33].

Several other Tau isoforms have a noteworthy impact on aggregation in AD. García-Escudero et al. recently discovered a new, human-specific truncated form of Tau generated by intron 12 retention [34]. This intron 12 retention generates a truncated Tau protein, followed by 18 extra amino acids, dramatically reducing this isoform’s ability to aggregate. While this Tau isoform exhibits similar biochemical properties and microtubule binding affinity, the variation seems to stabilize Tau, and is therefore considered to have a beneficial role. Interestingly, Cuadros et al. discovered that this form of Tau, called “W-Tau”, is reduced in AD brain at later stages of the disease. Another Tau isoform is the so-called “big-Tau”, a long-known high-molecular-weight isoform that contains an additional large exon termed 4a, which is sometimes without exon 6. Big-Tau expression and selective distribution is associated with neuronal development and regeneration [35,36], particularly in dorsal root ganglia. Chung et al. proposed that big-Tau expression is a feature of neurons that tend to be protected as AD progresses. In future studies, it would be prudent to study these isoforms in various regions of the AD-affected brain, considering that the occipital region often shows resilience to accumulation of AD pathology compared to the temporal and frontal regions, even within different portions of cortex. To our knowledge, the distribution of W-Tau or big-Tau isoforms, both relative to the DS-prodromal period or during DS-AD, has not been examined to date.

### 2.1. Posttranslational Modifications of Tau

The larger 4R Tau isoform contains 441 amino acid residues, and around 35 percent of 4R Tau isoforms undergo posttranslational modifications (PTMs) [37]. PTMs of Tau can occur at several different residues, including serine, threonine, tyrosine, lysine, arginine, asparagine, histidine, and cysteine [22]. PTMs at these sites include glycation, nitration, ubiquitination, and phosphorylation [26]. In pathological conditions, caused either by environmental or genetic factors, Tau can undergo multiple different PTMs and conformational changes, that can lead to Tau aggregation and tangle formations that can in turn develop into hallmarks of specific tauopathies [22,26,29]. Phosphorylation, which alters the physiological function and Tau protein affinity to microtubules, is the most studied PTM of the Tau protein. Phosphorylation can occur at 85 different sites on the Tau molecule, at the serine, tyrosine, or threonine sites [26], can modulate intracellular interactions significantly and lead to aggregating isoforms that can be toxic, both in vivo and in vitro [38]. The phosphorylation of a protein changes the charge, adding a negatively charged hydrophilic group, resulting in a hydrophilic rather than a hydrophobic protein molecule. More than 20 different kinases and phosphatases are known to regulate the phosphorylation of the Tau protein and are thought to underlie imbalances.

The complex relationship between Tau isoforms, phosphorylation and other PTMs at specific sites on the molecule provides crucial information that can be used to determine the cause of different tauopathies and develop novel and effective treatment paradigms. While Tau PTMs have been extensively studied in mouse models and in the *postmortem* brains of donors with AD and other tauopathies, there is still little research that examines how trisomy 21 (DS) affects the occurrence of Tau PTMs. Further, the complex changes associated with trisomy 21 may drive unique molecular effects on Tau solubility, intermolecular interactions, protein localization and degradation, in a manner that would make certain PTMs more likely to occur and perhaps more amenable to selective treatments. 

For example, DYRK1A, a dual kinase that can phosphorylate Tau at multiple positions [39], is upregulated in postmortem samples from brains with AD and particularly Down syndrome (DS), where a 50% increase is measured, due to having an extra copy of the gene on Chr. 21 [40,41,42]. Several strategies invoking DYRK1A inhibition improve cognitive function in mouse models of DS and have shown therapeutic benefits in young patients with DS in clinical trials [40,43]. However, the direct link between DYRK1A inhibition and suppression of Tau phosphorylation has only been demonstrated in fly and mouse models of AD [44,45,46]. Preliminary studies using spatial transcriptomics on paraffin embedded blocks suggest that there is an overexpression of Dyrk1A in specific hippocampal layers in *postmortem* human tissue (personal communication, Granholm) that is correlated with Tau-related alterations in the DS-AD brain, suggesting a definite link between p-Tau and Dyrk1A. While such a connection has been demonstrated in animal models [42,46] and *postmortem* protein-based studies, it has not previously been shown at the transcriptomic level.

### 2.2. The Role of RNA and DNA in Tau Aggregation

Tau-rich neurofibrillary tangles are complex protein-nucleic acid complexes [47,48] that grow intracellularly until the neuron is lost, often leaving behind a “ghost” tangle remnant. Several observations suggest that (1) Tau binds RNA [49,50], (2) RNA can promote insolubility of Tau [50,51], (3) RNA-binding proteins co-purify with Tau [52,53] and (4) RNA-specific dyes also stain Tau [54]. Recently, Lester et al. showed that Tau aggregates are enriched for small nuclear and small nucleolar RNAs, alter splicing speckles, and mislocalize nuclear splicing proteins [47] that all link components of the spliceosome. This evidence suggests that Tau sequesters RNA, leading to malfunction of cellular machinery, such as the spliceosome, resulting in cell death, which could help explain RNA processing defects seen in patients with tauopathies.

Other observations suggest that RNA induces unique Tau strains and seed competency. Zwierzchowski-Zarate et al. found that RNA homopolymers like PolyA could induce the formation of tau aggregates, indicating that defects in RNA surveillance could lead to neurodegenerative diseases [55]. Some of these PolyA-Tau aggregates in AD brain tissue were shown to be sensitive to RNase treatment. Interestingly, the origination source of RNA was important as human RNA potently and efficiently induced tau seed formation in a human HEK293T cell model, whereas RNA from other sources did not. This finding has implications for modeling Tau-RNA interactions as humanized Tauopathy mouse models may not successfully form the same RNA interfaces or aggregates seen in the human brain. Given that individuals with DS have significantly altered RNA complements, we wish to point out that Tau-RNA interactions have not been well studied in the context of Trisomy 21.

Another event that occurs with normal aging and AD is the accumulation of RNA and DNA quadruplexes [56], which have recently been shown to contribute to aging-related neuronal dysfunction and cell loss. G-Quadruplex (G4) DNA and RNA (RG4) are non-canonical secondary nucleic acid structures that have multiple roles in vital cellular processes, both in health and disease [56,57]. G4s are involved in regulating both DNA and RNA processes, including replication, transcription, and translation, and RG4s have been shown to particularly accumulate during cellular stress conditions [57]. A recent meta-analysis combining genome-wide association studies (GWAS) expanded the number of AD-risk loci, but most disease-associated variants reside in non-protein coding regions of the genome, such as in long non-coding (lnc) RNAs [58]. LncRNAs contribute to the pathogenesis of AD via modulating amyloid production, Tau hyperphosphorylation, mitochondrial dysfunction, oxidative stress, synaptic impairment and neuroinflammation, and have been thoroughly reviewed previously [59,60,61,62]. With lncRNA, several therapeutic approaches have been built around competing with endogenous RNA modulation [63], and determining whether or not various modulation technologies will be effective in physiological contexts remains a high-priority research agenda.

Few studies have focused on GWAS-derived risk loci in the context of DS, but it cannot be assumed that individuals with DS have similar risk loci to patients with late onset AD (LOAD). In the GWAS studies mentioned earlier, genetic mutations were found to be specific to G4s folded in proximal promoter regions of diverse sets of genes and pathways implicated in LOAD [64]. G4s are known to prevent protein aggregation via quadruplex-protein oligomerization [65], but few studies have tested this activity in the context of Tau and amyloid aggregation with AD. Our group has recently shown that RG4s accumulate in the hippocampal formation with aging and in patients with AD, and also demonstrated that intracellular accumulation of RG4s correlates significantly with NFTs and Braak stage [66]. These findings are exciting and could lead to a better understanding of the role of DNA and RNA secondary structures in the development of Tau pathology in AD and DS-AD. Further studies with mouse models of AD are warranted, since G4s are a prominent feature of mature neurons and are particularly prominent in the mouse hippocampus [67].

### 2.3. Local Tau Folding Patterns Confer Seed Competence

Posttranslational modifications of Tau (PTMs) also include truncation of the protein, which can occur at different molecular locations by various proteases in the cytosol. Proteolytic truncated pieces of Tau are highly prone to aggregation and contribute to progressive pathology in AD and DS–AD via site-specific phosphorylation, self-aggregation, and binding to hyperphosphorylated and oligomeric Tau [68,69]. It has recently been shown that truncated forms of Tau from the C-terminal have seeding capacity and spread via axonal pathways to different brain regions, causing local toxicity and neuronal death by, for example, affecting the autophagy system [70]. Truncated Tau protein also contributes to microtubule instability, potentially leading to synaptic dysfunction.

While PTMs, truncations, and RNA/DNA chaperone regulation all affect Tau aggregation, it was demonstrated by Diamond et al. [71] that the *cis* vs. *trans* isomeric configurations of specific Tau amino acids can have a significant impact on Tau’s soluble structure and seed capability. Specifically, a *trans*-proline configuration at P301 unfolds a local hairpin to expose the important aggregation motifs VQIINK or VQIVYK, which enhance self-assembly and template more significant seeding capacity [71]. In fact, *trans*-P301 appears to be one of the earliest forms of seed-competent Tau detected in a mouse model of AD-like tauopathy and was found to precede the formation of larger fibrillary assemblies in the brain [72]. This study suggests that the tertiary structure of Tau typically hides the parts of the protein that allow self-aggregation. The pathogenic form of Tau then exposes the parts of the molecule that allow it to aggregate, enabling self-assembly into oligomers and further into fibers, affording the protein prion-like capabilities.

Interestingly, antibodies raised in response to another *trans*-proline configuration, at P270 of the Tau molecule, can detect this isoform at more than 1000-fold higher levels in AD vs. non-AD brain samples, suggesting that local folding patterns are significantly disturbed in AD. The structural relationships remain of intense interest to researchers, as different sites within Tau seem to be associated with different tauopathies. In contrast, Qui et al. [73] recently discovered that the phosphorylated Thr231-Pro motif in Tau converts from *cis* to *trans* conformation in cells, animal models, and human AD brain [73,74,75]. However, *cis*, but not *trans*, P-tau is induced by hypoxic neuronal stress and after traumatic brain injury (TBI), a condition that P-Tau inclusions are a hallmark of [76].

These different research contributions support the notion that Tau seeding can originate from innate Tau structural properties, independent of the amyloidosis associated with AD. However, further studies are needed as AD-related amyloid burden is early and could itself augment Tau processes mentioned in the preceding sections, collectively leading to pathogenic forms of Tau being seeded.

## 3. Tauopathy Seeding and Its Spread in the DS Brain

Dr. Stanely Prusiner and his research group have minted AD as a “double-prion” disease, in acknowledgement of the prion-like activities conducted by both aggregating amyloid and Tau isoforms [77]. Interestingly, Condello and Prusiner examined prion activities in frozen brain tissue from individuals with DS of different ages by selectively precipitating Aβ and Tau from DS brain homogenates and using cellular bioassays to measure the number of prions. They discovered that while brain tissue from individuals with early-onset Alzheimer’s disease (EOAD) or LOAD exhibited reduced prion activities with age, this is not the case for DS brain tissue, where the levels of Aβ and Tau seeds increased with age [18]. These findings suggest a partially different seeding mechanism for Tau aggregates in DS, versus other forms of AD.

**Figure 1:**
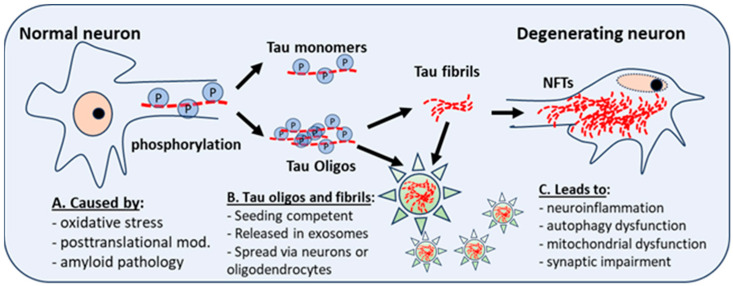
Posttranslational modifications and neuroinflammation or oxidative stress give rise to abnormal phosphorylation of Tau (P). This affects the stability of microtubules, and the development of Tau monomers and oligomers, which have aggregation potential and can form Tau fibrils, which aggregate into neurofibrillary tangles (NFTs), a hallmark of AD and DS-AD. Intra-neuronal NFT accumulation leads to neuroinflammation, autophagy dysfunction, and mitochondrial dysfunction. Tau fibrils and oligomers, seeding competent and secreted in exosomes (star), spread to other neurons and astrocytes, microglia, or oligodendrocytes. Oligos = oligomers.

As mentioned above, truncated, fibrous, and oligomeric Tau are seeding competent and can seed and spread, both in vivo and in vitro, when given the opportunity to propagate in a prion-like manner [32,78]. Recent studies have implicated fibrillar, truncated and oligomeric Tau in the seeding, aggregation, and propagation of Tau pathology in the brain [23,32,33,79,80,81]. Interestingly, oligomeric and fibrillar Tau appear to be equivalent in potency, in terms of seeding competency, and are both known to be taken up by local neurons after intracranial injection [82]. However, oligomeric Tau appears to drive a more potent glial response in the brain and a more rapid propagation of misfolded Tau to other brain regions [82] (see Figure 1 below).

### 3.1. P-Tau Spreading in Neurotransmitter Systems with AD

Noradrenergic neurons of the locus coeruleus (LC–NE; [83,84]), basal forebrain cholinergic neurons (BFCNs; [85,86,87,88]), and hippocampal neurons [89,90,91,92,93] are particularly vulnerable to dysfunction and/or degeneration in the brain of persons with DS. BFCNs are highly susceptible to degeneration in both DS–AD and AD and have crucial functions for learning and memory, both in human cohorts and animal models [87,94]. BFCNs have been studied in detail, in both DS–AD and AD, and the loss of these cells corresponds significantly with the decline in memory function in patients with dementia [94,95,96,97]. After early findings (in the 1970s and 1980s) that acetylcholinesterase inhibitors could slow the progression of memory loss in patients with AD [94,95,96,98,99,100], this became the most used anti-AD class of drugs to this day, despite these drugs not being disease-modifying and potentially producing severe and uncomfortable side effects [100]. However, choline esterase inhibitors have not been extensively used in patients with DS-related AD, due to side effects that might interfere with and contribute to comorbidities, even after a recent study suggested that patients with DS–AD could benefit from cholinesterase inhibitors [101]. Interestingly, recent work has shown that pre-tangle pathology (with an antibody that detects phosphorylated Tau at S422) coincided with the loss of staining of the p75 neurotrophic factor receptor (NTR) in the basal forebrain of patients with no cognitive impairment (NCI) or mild cognitive impairment (MCI); furthermore, these changes correlated with cognitive decline and AD neuropathology, confirming earlier studies that showed a connection between BFCN cell loss and Tau pathology [102]. On the basis of these findings, it is strongly suggested that Tau misfolding and phosphorylation drive cholinergic cell loss in the basal forebrain, both in AD and DS–AD.

LC–NE neurons degenerate prior to any other marked cell loss in the DS and AD brain [84,103]. Tau accumulates early in LC–NE neurons in AD, perhaps even decades before dementia symptoms; from there, prion-like spread of oligomers occurs, or filamentous Tau spreads from the LC to the forebrain and onto other brain regions [104,105,106]. There is a direct innervation of LC–NE neurons to BFCNs [107], potentially suggesting that early Tau pathology observed in BFCNs could be the result of spreading Tau “seeds” from the LC–NE to BFCNs, although this pathogenic potential still needs to be examined. Both Tau oligomers and Tau fibrils have seeding competency and can be secreted from neurons and also be taken up by other cells, including other neurons, astrocytes, microglia, or oligodendrocytes (see Figure 1 and [32,79,108,109]). However, the seeding competency of Tau, whether extracted from the DS brain or from DS–AD NDEs has not been previously examined.

### 3.2. Neuronal Exosomes Harbor Pathogenic Tau Seeds

**Figure 2:**
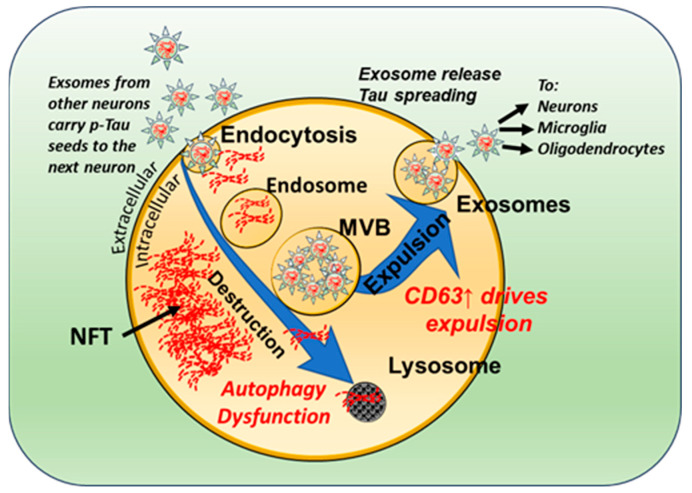
Schematic drawing showing alterations in the exosomal biogenic pathways in DS. Exosomal secretion is increased in individuals with DS, due to a reduced lysosomal/autophagy function and increased levels of the tetraspanin CD63. Endocytosis leads to the accumulation of misfolded Tau (oligomeric and/or fibrillar, red threads) intracellularly into NFTs. Exosomes containing the toxic Tau species can either undergo autophagy or be expelled. It has been shown that exosomes released from neurons can be taken up by other neurons and glial cells in the vicinity. Stars = exosomes; MVB = multivesicular bodies; NFT = neurofibrillary tangles.

A few years ago, Goetzl et al. [110,111,112] developed a new biomarker method, in which exosomes derived from neurons (NDEs, [110]) or astrocytes (ADEs, [111,112]) were purified from plasma or serum samples and biomarkers within their cargo, and then used to predict dementia decades before the onset of dementia [110]. We utilized this method and purified NDEs from plasma samples of individuals with DS at different ages, extending from childhood to older adults [20], and showed that NDEs from children with DS already contained AD-related p-Tau species at an early stage in life, compared to typically developing children. By using consecutive cycles of shear-induced fragmentation [25,28,78,113], we have shown that NDEs, isolated from DS plasma, contain seeding competent Tau conformers [21]. As discussed below, NDEs from DS–AD patients, when injected into the hippocampus of wild-type mice, gave rise to Tau pathology spreading to the mouse brain. The potential for misfolded Tau to spread from a DS–AD patient to the mouse brain had not been previously demonstrated. Other researchers have shown that toxic isoforms of Tau can be carried in exosomes and spread to other neurons, via the normal exosomal expulsion/uptake mechanisms (Figure 2, see also [21,114,115]).

In preceding sections, we identified that splicing isoforms, truncations and various PTMs significantly affect Tau seeding competency. There are many different Tau isoforms, but there are still major gaps in knowledge about which are transmitted via exosomal release. First, we do not know how pathogenic Tau seeds affect exosome biogenesis during AD (Figure 2). Chen et al. recently observed that Tau is readily taken up into the cell via endocytosis, followed by release into the cytosol when the endolysosomal system was compromised, where propagation of tau aggregation occurred [116]. Yan et al. revealed that increased exosome release counteracts endolysosomal dysfunction of Tau processing but increases the number of aggregates and the propagation of Tau [117]. In considering DS fibroblasts, Dr. Efrat Levi’s group showed that the elevated exosomal release depended on a unique tetraspanin called “cluster of differentiation” (CD)-63 (see Figure 2) and that the knockdown of CD63 function increased Tau seeding. In DS–AD, brain levels of CD63 are elevated [118], which could be one mechanism that is involved in adult DS brains expelling nearly 40% more NDEs than age-matched non-DS controls. We know that hallmark biomarkers used to classify the amyloid, tau and neurodegeneration (ATN) profile in plasma, including amyloid peptides, several P-Tau isoforms and neurofilament [119], are elevated in both AD and DS–AD [20,120,121], but there has been no systematic study that quantifies the other isoforms of Tau in this unique niche. Further, it remains unclear if other proteins co-transmit with Tau in neuronal exosomes that exacerbate Tau seeding. It does however seem that Tau “alone” can induce seeding as shown, for example, when it is isolated from exosomes [21] and even when tauopathy is negligible; thus, we hypothesize that the mechanism is involved in the seeding of Tau in the human brain.

### 3.3. Tau Seeds Impart a Bystander-Spreading Effect in the Brain

**Figure 3:**
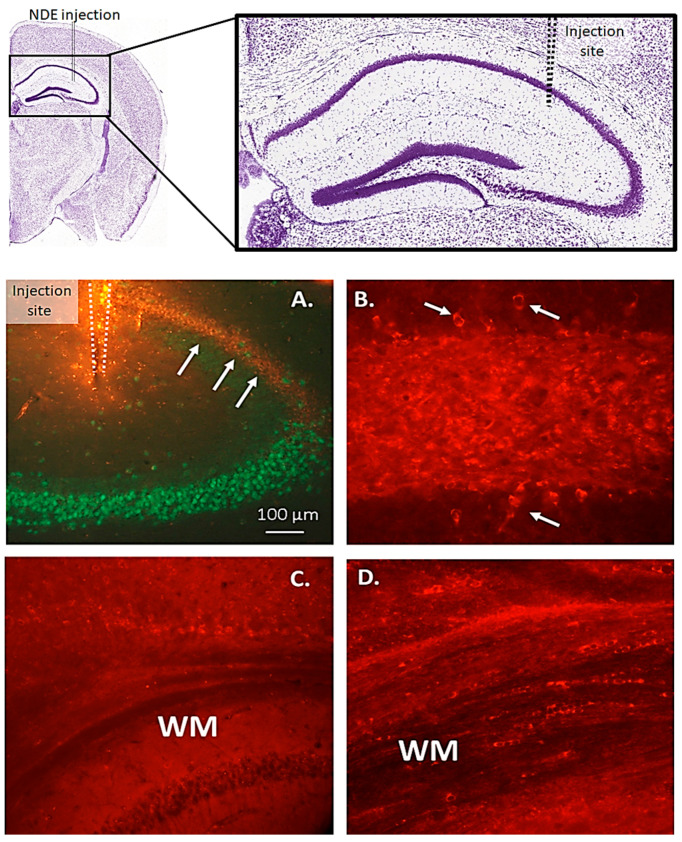
Stereotaxic injection of DS–AD neuron-derived exosomes (NDEs) into the dorsal hippocampus of WT mice gave rise to a spreading of p-Tau (S396), immunostaining along the CA1 pyramidal cell layer (**A**, arrows) to reach brain-wide distances from the injection site after several months. Within the hippocampal dentate gyrus (**B**), multiple flame-like neuronal inclusions of p-Tau (S396) i.r. could be seen one month after the injection (**B**, small white arrows). The NDE injection gave rise to a plexus of small cell bodies and fibers in the white matter (WM) that stained p-Tau (S396) (**D**) but not p-Tau (T231, **C**), suggesting that certain isoforms of p-Tau are more capable of seeding from neurons to oligodendrocytes in the white matter.

Since p-Tau and other forms of toxic Tau are part of exosome cargo and can be spread in the brain via exosomes (Figure 2 and [114]), we conducted experiments in which neuron-derived exosomes (NDEs) were purified from DS–AD plasma and injected into the dorsal hippocampus of a wild-type mouse (WT) with stereotaxic techniques (Figure 3 and [21]). Control experiments using exosome-specific antibodies and Nanosight technology, demonstrated the purity of the NDE injection and confirmed that only NDEs, and not other vesicles with different cellular origins, were injected [21].

In our studies, we observed a significant spreading of p-Tau (S396) immunoreactivity (i.r.) after the NDE injection in patients with DS–AD, but not controls. We saw no evidence of p-Tau (T231) i.r. spreading beyond the cells that were stained with this p-Tau isoform in the hippocampus. Double labeling with glial and neuronal stains revealed that most of the white matter cells stained with p-Tau (S396) antibodies were also co-labeled with Olig-2 and SOX-10, two stains that are specific for oligodendrocytes in the CNS. These findings suggest that NDEs from DS–AD patients can give rise to p-Tau (S396) seeding in the WT mouse brain and lead to Tau pathology spreading from neurons in the hippocampus to oligodendrocytes in the white matter and onto other brain regions, at least after NDE injections into the mouse brain [21].

These results are corroborated by Ferrer et al. [122], who recently published an interesting study that showed oligodendrocytes are highly involved in Tau spreading in the brain after brain homogenate injections from patients with different tauopathies, including AD, primary age-related tauopathy (PART), aging-related tau astrogliopathy (ARTAG) and globular glial tauopathy (GGT). After inoculation into the mouse brain, they reported that p-Tau aggregates were found in oligodendrocytes, along with neurons, to a greater degree than in other glial populations [122]. They also discovered that Tau seeding from the human brain gave rise to a response in mouse neurons, with increased levels of active Tau kinases, including p38 and ERK 1/2, suggesting that human misfolded Tau could give rise to active Tau phosphorylation of murine Tau [122]. Further, the seeding effect of pathogenic Tau seeds from AD brains has been shown to trigger Tau spread in macaque brain [123]. Thus, to date, misfolded Tau isoforms can spread as seeds between different species, and give rise to Tau pathology in the recipient brain, causing alterations in the conformation of the recipient Tau. Such seeding competency has not, as yet, been described for amyloid, which is another prion-like protein in the AD brain. However, it has recently been shown that amyloid can promote seeding capacity and the spreading of both Tau and alpha-synuclein in neurodegenerative disorders [124].

We do not yet know if the injections of DS-AD NDEs from human plasma into mouse hippocampus gave rise to murine Tau misfolding in neurons or oligodendrocytes in our studies, but it will be important for studies to address this in the future. We have also not seen other studies that investigate the seeding potency of p-Tau (S396) and compare it to other isoforms of p-Tau, and this is another subject that will also be of interest to future research. It is also possible that human tau mRNA transcript isoforms with differences in the predominance of 3R tau versus 4R tau may exhibit different seeding potencies and/or different morphological consequences in the host mouse brain, as some investigators have suggested [125]. Ferrer and his collaborators showed that Tau strains produce different patterns of active neuronal seeding, which also depend on the host Tau [122,125]. These studies could have a significant bearing on the pathological cascade in the brain caused by Tau misfolding and seeding and lead to better and more targeted drugs that could stop the progression of dementia in several different tauopathies.

## 4. Tau Biomarkers in Biofluids

Individuals with DS develop dementia and AD pathology at a variable age, but earlier than in LOAD and perhaps earlier than in EOAD. Given that as much as 90% of individuals with DS develop AD-like dementia by the fifth decade of life [126], one might hypothesize that Tau seeding and spread within the DS brain may occur decades earlier. Recent improvements in measuring P-Tau by immunoassays, seeding assays and Positron Emission tomography (PET) have enabled this hypothesis to be tested and led to several breakthroughs, while producing new questions about when Tau gains seeding “prion-like” capacities.

### 4.1. Ultrasensitive Immunoassays

With the recent development of new sensitive assays such as the single molecule array (Simoa [127]), investigators are now capable of measuring even minute amounts of biomarkers in plasma in different neurological conditions. We performed a study focused on exosome biomarkers in individuals with DS, showing increased amyloid exosomal levels already in early childhood, which increased through adulthood [19]. However, we also unexpectedly found an increase in p-Tau (S396 and T181) when comparing all DS and all control participants at an early age, and noted an increase of p-Tau with age (T181) [20]. These findings suggested that misfolding and phosphorylation of Tau may be an early event in DS and could be used to predict the onset of dementia, and also improve treatment efficacy for this population. Measuring biomarkers in exosomes proved to be a sensitive and possibly less variable assay of dementia-related biomarkers than when the same biomarkers were applied in plasma, which was due to the relatively low levels of AD-related proteins observed in plasma.

In engaging with older adults with DS, Alzheimer Biomarkers Consortium—Down Syndrome (ABC–DS) demonstrated early changes in persons with DS by using different p-Tau isoforms in plasma [128]. The Petersen et al. group [128] studied more than 300 participants with DS at different ages, showing that plasma levels of total Tau and neurofilament light (NfL) were highly predictive of both AD pathology and clinical status in those with DS at different ages. In another recent biomarker study for the European DS clinical network Horizon 21, Carmona and collaborators examined neurofilament light (NfL) [129], and found that NfL plasma levels had excellent diagnostic performance and a highly similar temporal distribution of change, compared to that seen in autosomal dominant AD [129]. Phospho-Tau biomarkers in plasma have also been examined by using ultrasensitive methods [130,131]. And Plasma p-Tau (T217), glial fibrillary acidic protein (GFAP), amyloid beta peptides 42 and 40 (Aβ42/Aβ40), NfL, and total Tau (t-Tau) were assayed. The study, which included 300 participants with DS and 37 non-DS siblings, found that higher p-Tau (T217) levels and no other biomarkers were associated with worse performance in the DS mental status examination and cued recall test, suggesting that plasma p-Tau (T217) is an accurate blood-based biomarker of both Tau and Aβ pathological brain changes in DS that could be used to include individuals with DS in AD clinical trials, especially when combined with age as a covariate [130]. Since plasma levels of AD-related pathology are easier to accomplish in the clinic than in NDE assessments, this newly developed measure may lend itself better to large clinical studies than to more cumbersome exosomal biomarker studies [20,132].

### 4.2. Tau Seeding and Aggregation Assays

Promising techniques have been developed for the quantitation of Tau seeding activity in human biofluid. Holme et al. (2014) developed a cell-based assay, where a Tau-containing biosample is incubated with a cell line overexpressing tau linked to a fluorescent protein [108]. Upon aggregation, energy transfers between fluorescent proteins (FRET) allowed for the detection of seeding capacity signals using flow cytometry. Using this FRET method, robust Tau seeding activity could be observed one month before histopathological stains showed NFTs, suggesting that tau seeding is an early signature of tauopathy. Cell-free assays have been developed by real-time quaking-induced conversion (RT-QuIC) to measure prion seeding [133] and, more recently, Tau seeding [134]. Tau seed-containing material from biofluids or brain samples is incubated with recombinant tau substrate and thioflavin T in optimized conditions. When RT-QuIC is used, seeding-competent material induces the aggregation of the substrate, which generates a fluorescent signal.

Jin et al. (2022) recently developed a Tau seeding activity assay using truncated HA-tagged Tau151-391 peptide and cellular transformation [135]. Cells transiently express the HA-tagged Tau151-391 peptide, which is readily captured and aggregated by oligomeric Tau derived from *postmortem* AD brain samples. The captured Tau is then quantified using traditional immunoblot methods. They employed these assays on AD and DS brain samples in two different studies [136] and discovered that only brain extracts from AD or DS brain samples contained hyperphosphorylated Tau seeded Tau aggregation in cultured cells. Interestingly, Tau extracts of DS corpus callosum showed low tau seeding activity, and no detectable tau seeding activity was observed in DS cerebellar cortex. Again, Tau seeding ability was found to be highly correlated with phosphorylation, but the group did not study the effects of other Tau PTMs in this assay.

### 4.3. Tau Binding Studies Using Positron Emission Tomography (PET) Ligands

Tau PET imaging is emerging as an important clinical tool for early diagnosis of AD and other tauopathies, as p-Tau appears to correlate more closely than amyloid with symptomatic dementia progression. In the last ten years, multiple Tau PET ligands have been developed. Tau-specific ligands for use in PET include first-generation ligands and, consequently, second-generation ligands (see Leuzy et al. [137]). Several of these Tau PET ligands have been tested in patients with DS and AD including, for example, the [18F]-AV-1451 Tau PET ligand [138]. Rafii et al. demonstrated that amyloid-negative participants who were DS imaged were all Tau-negative, and that both amyloid and Tau burden correlated with age [138]. They also found that Tau binding in the brain correlated significantly with cognitive decline, suggesting that this clinical measure can be used to predict the onset or progression of dementia in DS-AD. Recently, Dr. Bradley Christian and his research group have performed longitudinal PET analyses in a DS cohort with the aim of better defining a timeline for the progression of amyloid and Tau burden through the conventional Braak stages [139,140,141]. By using PET scans, they revealed early and rapid Tau elevation following the onset of amyloid-positive PET imaging. While Tau elevation is highly variable in AD cohorts, individuals with DS displayed uniform increases in Tau within 2.5 years of the onset of amyloid binding in the brain. Although very early in life, the spatial progression of neurofibrillary Tau tangles and paired helical filament Tau in DS closely follows the hierarchical staging pattern outlined by Braak and Braak for LOAD studies [142]. This pattern has been well described by Davidson et al., who observe that Tau pathology usually commences after 35-years-of-age, in the entorhinal cortex, hippocampal formation, and in subcortical brainstem regions such as the locus coeruleus and dorsal raphe nucleus. Later, Tau pathology spreads throughout the neocortex, sparing the occipital lobes until the mid-50 years of age [18]. Although this pattern is generally recognized in individuals with DS, there are reports showing that dysregulation of Tau phosphorylation can occur quite early in life in those with DS [143], which is something that cannot be detected by Tau PET imaging, which serves to demonstrate the need for detailed *postmortem* tissue studies.

To further quantify the binding of Tau PET ligands in specific brain regions, we performed an autoradiographic binding study of Tau PET ligand binding in *postmortem,* fixed and paraffin-embedded brain tissue sections from individuals that received a neuropathological diagnosis of either LOAD, EOAD, or DS-AD, and compared them against age-matched controls [144]. Interestingly, we found that the binding of both the first generation (THK5117) and the second generation (MK6240) Tau PET ligand exhibited a significant increase in frontal cortex (middle frontal gyrus) in DS–AD *postmortem* cases, compared to both EOAD and LOAD cases. In addition, autoradiographic binding of both of these Tau ligands correlated significantly with AT8 p-Tau immunostaining of adjacent sections, strongly suggesting that on-target binding was more prevalent than off-target binding, at least in these fixed and paraffin-embedded sections [144]. Other studies have suggested that in fresh frozen materials and in vivo, there is off-target binding of Tau ligands to, for example, monoamine oxidase B (MAO-B) [145], which did not appear to be the case in our study using fixed materials. Investigators have identified a binding site for all the Tau tracers on MAO-B [145], which could make clinical studies difficult to undertake unless a better understanding of off-target binding is first achieved. However, it was reported by Murugan et al [145] that a second-generation Tau PET ligand, MK6240, has a lower affinity for MAO-B than the first-generation tracers. Nevertheless, in our binding study, we found a significant correlation between p-Tau immunostaining (AT8 antibodies), with both the first and second-generation Tau ligands used in this study [144], suggesting less off-target binding in fixed tissues than what was previously reported in vivo.

## 5. Discussion

Tau pathology is an important component of AD research but also carries a significant pathological burden in other tauopathies [146]. Tauopathies can be clinically classified on the basis of a range of symptoms related to cognition, behavior, mental health, motor function, language disabilities and non-specific amnestic or executive symptoms. Pathologically, tauopathies can be classified based on the predominant Tau isoforms and/or phosphorylation sites that are present in inclusion bodies or cytoskeletal fibrils, including the ratio of 3R:4R and truncation of the Tau protein at specific sites [146]. The most recently classified tauopathy is chronic traumatic encephalopathy (CTE), which is pathologically classified by the accumulation of Tau inclusions, particularly in the sulci [30]. Recent work has shown that repeated traumatic brain injuries (TBIs) in mouse or rat brains give rise to an accumulation of cis-phosphorylated Tau [147] as well as Tau oligomers in CSF or brain tissue. However, the specific form and inclusion pattern of Tau appears to be significantly different in different forms of tauopathy [22,70].

In studying individuals with DS and AD (DS–AD), Condello and collaborators found a significant difference in prion activity, compared to other forms of AD, with DS brains showing a continuously increasing prion activity of Tau and amyloid with age; this is contrary to the reduced prion activity of these proteins that is observed in the brains of patients with LOAD or EOAD [18]. This would suggest that DS brains contain a particularly violent form of Tau seeds and this may, at least partially, explain the early onset of AD pathology and dementia in this population [5].

Our binding studies using Tau tracers suggested that the frontal cortex was more affected in terms of Tau binding in DS–AD, compared to LOAD or EOAD [144], which is potentially due to the developmental detriments in frontal cortex organization that have been reported for children with DS, including hypoconnectivity [148], reduced neuronal numbers and brain volume [149], and underdeveloped neuronal migration in the frontal cortex [97,150]. This could, compared to the frontal cortex in typically developing individuals, contribute to the relative vulnerability of the frontal cortex to Tau and amyloid pathology as the individual with DS gets older.

The fact that the gene that encodes amyloid precursor protein (*APP*) is located on Chr. 21 [151] can also contribute to the vulnerability of individuals with DS to AD pathology. Since APP in the fetal brain is highly involved in neurogenesis, neuronal differentiation and synaptogenesis during neurotypical development [152,153], an overproduction of amyloid could lead to a loss of normal APP function during development—hence, a delayed migration of neurons in the cortical plate and/or disturbance in synaptic development. The Tau protein is also directly involved in neural and synaptic development. Studies have shown that human phosphorylated fetal Tau (3R, containing exon 0) is found in the distal portions of cortical growth cones [154] and contributes to axonal growth and maturation [154]. This isoform of p-Tau does not generate aggregates in the brain but is altered early in the maturation of the DS brain [143], potentially also contributing to reduced maturation, particularly of cortical regions. Thus, although we know that aberrant prenatal brain development in individuals with DS can disrupt the function and structure of cortical areas during adulthood and aging, further studies are needed in order to design appropriate interventions that can halt or reduce the impact of AD pathology in individuals with DS.

Anti-Tau monoclonal antibodies have enabled novel immunotherapy strategies to treat AD [155]. However, four of these, including gosuranemab, tilavonemab, semorinemab, and zagotenemab, have all failed in phase II clinical trials for either AD or PSP [156]. Despite these early setbacks, the emergence of new AD-relevant Tau isoforms mentioned in this review could aid in developing new targets for intervention. We want to highlight that all Tau monoclonal antibodies must be evaluated for adequate clearance of Tau seeds, since seed-competent isoforms may conformationally resist antibody binding. The field is rapidly progressing and will undoubtedly test new theories related to tauopathy in upcoming clinical trials. Adults with Down syndrome are participants ideally suited to clinical trials targeting prevention, yet have historically been excluded from trials. However, efforts are underway to provide an optimal infrastructure, centralized research resources and shared expertise that will accelerate the development of effective interventions for individuals with DS, including the “Alzheimer’s Clinical Trials Consortium—Down Syndrome” and the “Down Syndrome Biobank Consortium” [157,158].

## 6. Conclusions

Tau misfolding and aggregation play a major role in AD pathology as well as in DS–AD pathology. This review demonstrates recent work focused on understanding the role of NFTs and monomeric/oligomeric Tau in the prion-like spreading of Tau pathology in the human brain, and does so with the aim of coming up with novel treatment paradigms for this debilitating and early onset AD form that occurs in those with DS. This review sheds light on the underpinnings of Tau pathology in DS–AD and demonstrates the central role that misfolding of the Tau protein plays in this pathological process.

## Data Availability

Data pertaining to this review are available via the DSBC website (https://medschool.cuanschutz.edu/neurosurgery/research-and-innovation/services/down-syndrome-biobank), or via our previously published work, which is indicated in the publication list.
